# *Helicobacter pylori* virulence genes in the five largest islands of Indonesia

**DOI:** 10.1186/s13099-015-0072-2

**Published:** 2015-10-05

**Authors:** Muhammad Miftahussurur, Ari Fahrial Syam, Dadang Makmun, Iswan Abbas Nusi, Lukman Hakim Zein, Zulkhairi, Fardah Akil, Willi Brodus Uswan, David Simanjuntak, Tomohisa Uchida, Pangestu Adi, Amanda Pitarini Utari, Yudith Annisa Ayu Rezkitha, Phawinee Subsomwong, Nasronudin, Yoshio Yamaoka

**Affiliations:** Department of Environmental and Preventive Medicine, Oita University Faculty of Medicine, Yufu, 879-5593 Japan; Gastroentero-Hepatology Division, Department of Internal Medicine, Airlangga University Faculty of Medicine, Surabaya, 60131 Indonesia; Institute of Tropical Disease, Airlangga University, Surabaya, 60115 Indonesia; Division of Gastroenterology, Department of Internal Medicine, Faculty of Medicine, University of Indonesia, Jakarta, 10430 Indonesia; Division of Gastroentero-Hepatology, Department of Internal Medicine, Faculty of Medicine, University of Sumatera Utara, Medan, 20136 Indonesia; Department of Internal Medicine, Faculty of Medicine, Center of Gastroentero-Hepatology, Hasanuddin University, Makassar, 90245 Indonesia; Department of Internal Medicine, Santo Antonius Hospital, Pontianak, 78115 Indonesia; Department of Internal Medicine, Yowari Hospital, Jayapura, 99352 Indonesia; Department of Molecular Pathology, Oita University Faculty of Medicine, Yufu, 879-5593 Japan; Department of Gastroenterology and Hepatology, Baylor College of Medicine and Michael DeBakey Veterans Affairs Medical Center, Houston, TX 77030 USA

**Keywords:** *Helicobacter pylori*, Indonesia, Virulence factors

## Abstract

**Background:**

It remains unclear whether the low incidence of gastric cancer in Indonesia is due to low infection rates only or is also related to low *Helicobacter pylori* pathogenicity. We collected *H. pylori* strains from the five largest islands in Indonesia and evaluated genetic virulence factors.

**Methods:**

The genotypes of *H. pylori* virulence factors were determined by polymerase chain reaction (PCR)-based sequencing. Histological severity of the gastric mucosa was classified into 4 grades, according to the updated Sydney system.

**Results:**

A total of 44 strains were analyzed. Forty-three (97.7 %) were *cagA*-positive: 26 (60.5 %) were East-Asian-type-*cagA*, 9 (20.9 %) were Western-type-*cagA,* and 8 (18.6 %) were novel ABB-type, most of which were obtained from Papuan. EPIYT sequences were more prevalent than EPIYA sequences (P = 0.01) in the EPIYA-B motif of all types of cagA. The majority of *cagA*-positive strains (48.8 %, 21/43) had a 6-bp deletion in the first pre-EPIYA region. Subjects infected with East-Asian-type-*cagA* strains with a 6-bp deletion had significantly lower inflammation and atrophy scores in the corpus than those infected with Western-type-*cagA* strains (both P = 0.02). In total, 70.4 % of strains possessed the *vacA* s1m1 genotype and 29.5 % were m2. All strains from peptic ulcer patients were of the *iceA1* genotype, which occurred at a significantly higher proportion in peptic ulcer patients than that in gastritis patients (55.3 %, P = 0.04). The double positive genotype of *jhp0562*/*β*-*(1,3)galT* was predominant (28/44, 63.6 %), and subjects infected with this type had significantly higher inflammation scores in the corpus than those with the *jhp0562* negative/*β*-*(1,3)galT* positive genotype (mean [median]; 1.43 [1] vs. 0.83 [1], P = 0.04). There were significant differences in *cagA* and pre-EPIYA *cagA* type, *oipA* status, and *jhp0562*/*β*-*(1,3)galT* type among different ethnic groups (P < 0.05).

**Conclusions:**

In addition to a low *H. pylori* infection rate, the low incidence of gastric cancer in Indonesia might be attributed to less virulent genotypes in predominant strains, which are characterized by the East-Asian-type-*cagA* with a 6-bp deletion and EPIYT motif, a high proportion of m2, *dupA* negative or short type *dupA*, and the *jhp0562*/*β*-*(1,3)galT* double positive genotype.

## Background

*Helicobacter pylori* infection remains latent in the majority of infected patients, and only a minority of individuals with *H. pylori* infection ever develop severe disease [1]. Moreover, the differences in *H. pylori* infection rates cannot adequately explain differences in the incidence of gastric cancer in the world. Therefore, in addition to host and environmental factors, differences in the incidence of gastric cancer irrespective of *H. pylori* infection rate can be explained by differences in virulence factors [2]. Several genes have been proposed as possible virulence determinants: *cagA*, *vacA*, *oipA*, *iceA*, *dupA*, *jhp0562,* and *β*-*(1,3)galT* [2, 3].

*cagA* is the major *H. pylori* virulence factor. The sequence of the second repeat region was found to differ considerably between East-Asian-type-*cagA* and Western-type-*cagA*. Each CagA is assigned to a sequence type consisting of the names of the EPIYA segments in its sequence (that is, ABC, ABCC, or ABCCC for Western-type- and ABD for East-Asian-type-*cagA*). East-Asian-type-*cagA* has a higher binding affinity for the Src homology-2 domain-containing phosphatase 2 (SHP2), resulting in a higher risk of peptic ulcer and/or gastric cancer than Western-type-*cagA* [4–7]. The pre-EPIYA region of *cagA*, located about 300-bp upstream of the first EPIYA motif, has also been investigated. Alignment of these sequences revealed that a 39-bp deletion was present in most strains isolated from East Asia, but was absent in most strains from Western countries (no deletion) [8].

*vacA,* the second major *H. pylori* virulence factor, produces variations in the vacuolating activity of *H. pylori* strains. In general, the *vacA* s1m1 strain produces a large amount of toxin with high vacuolating activity in gastric epithelial cells, while the s1m2 strain produces moderate amounts of toxin, and the s2m2 strain produces low or undetectable amounts of toxin [9]. *cagA* status is linked to the *vacA* s region type, and it is further closely linked to the presence of the *oipA* “on” status, which is a virulence factor coding an outer membrane protein [10, 11]. Previous studies demonstrated that almost all *H. pylori* strains circulating in Japan were extremely virulent, irrespective of clinical outcomes, and harbored the following genotype: East-Asian-type-*cagA*, *vacA* s1, and the *oipA* “on” status [11, 12].

An initial series of studies showed that *iceA* has 2 main allelic variants: *iceA1* and *iceA2* [10, 13]. The expression of *iceA1* was upregulated on contact between *H. pylori* and human epithelial cells, and the *iceA1* genotype was associated with enhanced mucosal interleukin (IL)-8 expression and acute antral inflammation [13, 14]. *dupA,* the first genetic factor of *H. pylori* to be characterized, was reported to be associated with a differential susceptibility to duodenal ulcer (DU) and gastric cancer [15]. Additionally, a number of recent studies have indicated that *jhp0562* and *β*-*(1,3)galT* were associated with the development of peptic ulcers [3, 16]. Our previous study indicated that in the US population, the absence of *β*-*(1,3)galT* was an independent factor for differentiating DU and gastric ulcer (GU) from gastritis [10]. Together with other virulence factors, *jhp0562* and *β*-*(1,3)galT* might be predictors of severe clinical outcomes of *H. pylori* infection, as well as of gastric cancer.

Indonesia is a country in Southeast Asia with low risk of gastric cancer; it is an archipelago with a multi-ethnic society. The age-standardized incidence of gastric cancer in Indonesia was reported to be 2.8/100,000, which is relatively low among Asian countries (available from the International Agency for Research on Cancer; GLOBOCAN2012, http://globocan.iarc.fr/). In March 2013, there were only 313 hospitals providing GI endoscopy services in Indonesia, and most of them were located on the island of Java [17]. Moreover, many patients with dyspepsia are not covered by the Indonesian health insurance system; therefore, it is difficult for them to undergo endoscopy. Our previous study using 5 different diagnostic methods confirmed that the prevalence of *H. pylori* infection in Surabaya (Java island) was low (only 11.5 %) [18]. We also found a low prevalence of *H. pylori* infection in a minor group of North Sulawesi; the prevalence was only 14.3 % for adults and 3.8 % for children [19]. However, it remains unclear whether the low incidence of gastric cancer in Indonesia is due to low infection rates only or also owing to low *H. pylori* pathogenicity. In this study, we collected *H. pylori* strains from the five largest islands in Indonesia and evaluated genetic virulence factors.

## Results

### Patients and *H. pylori*

From January 2014 to June 2015, we recruited a total 311 patients with dyspeptic symptoms (170 female and 141 male; mean age of 47.8 ± 14.6 years; range, 17–80 years) from several ethnicities in the 5 largest islands in Indonesia, including Jakarta and Surabaya (Java island), Jayapura (Papua island), Makassar (Sulawesi island), Pontianak (Borneo island), and Medan (Sumatera island). Among 311 patients, 180 (57.9 %) showed no gastric activity, inflammation, or atrophy, neither in the antrum nor in the corpus, by histological examination; these patients were considered to be the normal group. All subjects in the normal group were negative for *H. pylori* infection. Among the remaining 131 patients with some histological changes (activity, inflammation and/or atrophy), 44 (33.6 %) were positive for *H. pylori.*

Even though we obtained a large number of samples, only 39 strains could be isolated. We therefore decided to add 5 strains isolated in Surabaya (Java island) with corresponding histological information that had already been evaluated by the same pathologist (UT). These were isolated from patients with the following ethnicities: Javanese (n = 1), Floresnese (n = 2), and Chinese Indonesian (n = 2) [18]. Overall, a total of 44 strains (38 from patients with gastritis, 5 with GU, and 1 with DU) were included in the final analysis. Twenty-four strains were isolated from males (age range, 25–77 years; mean age, 49.4 years) and 20 from females (age range, 26–67 years; mean age, 47.4 years). The strains were from patients with the following ethnicities: 16 Batak, 9 Papuans, 5 Buginese, 7 Chinese Indonesian, 3 Floresnese, 2 Javanese, and 2 Dayak. Although the number of samples was not sufficient for statistically significant conclusions, we found that the Buginese, Dayak, and Papuan had a tendency toward higher inflammation in the antrum than the Floresnese (mean [median]: 2.00 [2], 2.5 [2.5], 2.0 [2.0] vs. 1.0 [1], P = 0.05).

### Virulence genes of Indonesian strains and histology

**Fig. 1:**
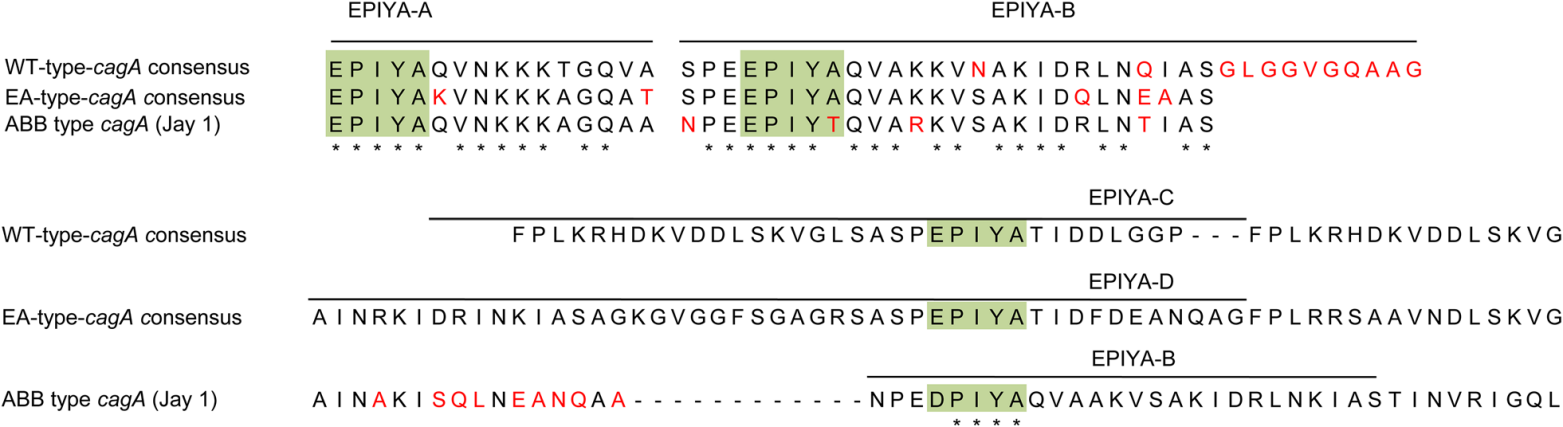
Sequence analysis of CagA structural polymorphisms in Indonesian strains. Eight strains had ABB type; most were Papuan, which was very rare in other countries. Sequences of the first and second segment B in the ABB type were similar to segment B in East-Asian-type-*cagA*; however, the second segment B contained a trace of the segment D component of East-Asian-type-*cagA*. The *star symbol* indicates the sequence similarities among all *cagA* types. In contrast, the *red color* emphasizes sequence differences

In total, 43 of 44 strains possessed the *cagA* gene (97.7 %). Sequence analyses revealed that 23 strains were of the ABD type and 3 were of the AABD type, which were both considered East-Asian-type-*cagA* (26/43, 60.5 %). Western-type-*cagA* (ABC, ABCC, BC, B) was found in 20.9 % (9/43) of isolates. One strain with B type was regarded as Western-type-*cagA* based on the sequence similarity of B-segments with Western-type-*cagA.* Interestingly, 8 strains had ABB type (18.6 %), which is very rare in other countries. Sequences of both B segments in the ABB type were different from those of East-Asian-type- or Western-type-*cagA* (Fig. 1). Therefore, we classified the ABB type as an independent group.

Sequence analyses of the 300 bp upstream of the first EPIYA motif (EPIYA-A) revealed that the predominant pre-EPIYA type contained a 6-bp deletion (48.8 %, 21/43), which is also very rare in other countries (Table 1). Eleven (25.6 %) strains contained an 18-bp deletion, which is typically observed in Vietnamese strains [8], and 3 strains contained a 39-bp deletion typically observed in East Asian countries. The remaining 8 strains contained no deletion, which is typically observed in Western countries. Only 1 Western-type-*cagA* was not classified as containing no deletion. For East-Asian-type-*cagA* strains, the predominant pre-EPIYA type contained a 6-bp deletion (80.8 %, 21/26). All ABB types contained an 18-bp deletion (Table 1).

**Table 1:** Association between *cagA* type and pre-EPIYA *cagA* type

*cagA* type	n	Pre-EPIYA *cagA* type			
		6-bp deletion	18-bp deletion	39-bp deletion	No deletion
East-Asian-type-*cagA*	26	21 (80.8 %)	2 (7.6 %)	3 (11.5 %)	0 (0.0 %)
Western-type-*cagA*	9	0 (0.0 %)	1 (11.1 %)	0 (0.0 %)	8 (88.9 %)
ABB type	8	0 (0.0 %)	8 (100.0 %)	0 (0.0 %)	0 (0.0 %)
Total	43	21 (48.8 %)	11 (25.6 %)	3 (7.0 %)	8 (18.6 %)

Histological analysis showed that inflammation and atrophy scores in the corpus were significantly higher in subjects infected with Western-type-*cagA* strains than those with East-Asian-type-*cagA* strains (mean [median]; 1.88 [1] vs. 1.23 [1], P = 0.03 and 1.00 [1] vs. 0.42 [0], P = 0.04, respectively) (Table 2), a result that opposes the current consensus. However when analyzed in detail, only subjects infected with strains possessing East-Asian-type-*cagA* containing the 6-bp deletion had significantly lower inflammation and atrophy scores in the corpus than those possessing Western-type-*cagA* (1.88 [1] vs. 1.19 [1], P = 0.02 and 1.00 [1] vs. 0.33 [0], P = 0.02, respectively) (Table 2). There were no significant differences in inflammation between subjects infected with strains possessing East-Asian-type-*cagA* containing the 18- or 39-bp deletions, or the ABB type with Western-type-*cagA* (all P > 0.05).

**Table 2:** Histological scores according to *cagA* type and pre-EPIYA *cagA* type

	Western-type-*cagA*	East-Asian-type-*cagA*			ABB type
		All	6-bp deletion	No 6-bp deletion	
n	9	26	21	5	8
Antrum					
Activity	1.63 (1.5)	1.15 (1)	1.19 (1)	1.50 (1.5)	1.63 (1.5)
Inflammation	1.88 (2)	1.73 (2)	1.76 (2)	1.60 (1)	2.00 (2)
Atrophy	1.25 (1)	1.04 (1)	1.05 (1)	1.00 (1)	1.13 (1)
Intestinal metaplasia	0.13 (0)	0.00 (0)	0.00 (0)	0.00 (0)	0.00 (0)
Corpus					
Activity	0.88 (1)	0.77 (0)	0.76 (1)	0.80 (1)	0.75 (1)
Inflammation	1.88 (2)	1.23 (1)*	1.19 (1)*	1.40 (1)	1.63 (2)
Atrophy	1.00 (1)	0.42 (0)*	0.33 (0)*	0.80 (1)	0.88 (1)
Intestinal metaplasia	0.25 (0)	0.00 (0)	0.00 (0)	0.00 (0)	0.00 (0)

Histology data are presented as mean (median)

* P < 0.05 compared to Western-type-*cagA*

The EPIYA motifs in these strains were also evaluated (Table 3). We obtained five types of EPIYA or EPIYA-like sequences. In total, 127 EPIYA motifs were obtained from the 43 CagAs. The 2 most common 5 amino acid EPIYA motifs were EPIYA (89/127, 70.1 %) and EPIYT (25.2 %), in agreement with our previous studies [20, 21]. The EPIYA-B motif displayed the biggest change in five amino acids, and EPIYT was more prevalent than EPIYA in all types of CagA (Table 3), not only in Western-type-*cagA,* which was reported previously [20, 21]. When we analyzed the first EPIYA-B motif, we found that subjects infected with strains with EPIYT sequences had higher inflammation in the antrum than those with EPIYA sequences (2.03 [2] vs. 1.27 [1], P = 0.01). Further, when we analyzed the first EPIYA-B motif only in East-Asian-type-*cagA*, we found that subjects infected with strains with EPIYT sequences had higher activity in the antrum than those with EPIYA sequences (1.35 [1] vs. 0.5 [0.5], P = 0.03). There were no significant differences in histological scores for EPIYT motifs in the ABB type and East-Asian-type-*cagA*.

**Table 3:** Frequencies of EPIYA motifs in Indonesia strains

Type	All motifs	No.	A motif	No	B motif	No	C or D motif	No
	EPIYA	89	EPIYA	44	EPIYA	11	EPIYA	34
	EPIYT	32			EPIYT	32		
	DPIYA	1			DPIYA	1		
	DPIYT	4			DPIYT	4		
	EPVYT	1			EPVYT	1		
Total		127						
ABD-type *cagA*	EPIYA	62	EPIYA	29	EPIYA	7	EPIYA	26
	EPIYT	19			EPIYT	19		
Total		81						
ABC-type *cagA*	EPIYA	18	EPIYA	7	EPIYA	3	EPIYA	8
	EPIYT	5			EPIYT	5		
	EPVYT	1			EPVYT	1		
Total		24						
ABB-type *cagA*	EPIYA	9	EPIYA	8	EPIYA	1	EPIYA	0
	EPIYT	8			EPIYT	8		
	DPIYT	4			DPIYT	4		
	DPIYA	1			DPIYA	1		
Total		22						

The predominant genotype for *vacA* was s1 (43/44, 97.7 %), whereas the *vacA* m2 genotype was found in 29.5 % (13/44), and the *vacA* s2 genotype was found in only 2.3 % (1/44) of isolates. For the combined *vacA* s and m regions, the s1m1, s1m2, and s2m2 were found in 70.4 % (31/44), 27.3 % (12/44), and 2.3 % (1/44) of isolates, respectively.

The *iceA1* genotype was predominant (61.4 %, 27/44), and 36.4 % of isolates were of the *iceA2* genotype (16/44). The remaining 1 isolate was positive for both *iceA1* and *iceA2* (*iceA1/iceA2* mixed). Interestingly, all strains from peptic ulcer patients (n = 6) were of the *iceA1* type; the *iceA1* type occurred more frequently in peptic ulcer patients than in gastritis patients (55.3 % (21/38), P = 0.04).

The *oipA* “on” status was predominant in Indonesian strains (40/44, 90.9 %), and the remaining strains were considered “off.” All peptic ulcer patients were *dupA* negative. Only 3 (6.8 %) strains were *dupA* positive (all were from gastritis patients) and none of them were the intact long-type *dupA*, a strong *dupA* virulence marker for severe outcomes [22]. There were no significant differences between *vacA*, *iceA*, *oipA*, and *dupA* status with gastric mucosal status (all P > 0.05).

The *jhp0562*/*β*-*(1,3)galT* double positive was the predominant type (28/44, 63.6 %), followed by *jhp0562* positive/*β*-*(1,3)galT* negative, and *jhp0562* negative/*β*-*(1,3)galT* positive (10/44 (22.7 %) and 6/44 (13.6 %), respectively). Histological analysis showed that inflammation scores in the corpus were significantly higher in subjects infected with strains containing the *jhp0562*/*β*-*(1,3)galT* double positive genotype than in those with *jhp0562* negative/*β*-*(1,3)galT* positive [mean (median); 1.43 (1) vs. 0.83 (1), P = 0.04], but not significantly different from those with *jhp0562* positive/*β*-*(1,3)galT* negative (P = 0.47).

### Virulence genes and ethnics groups

The association between virulence genes and ethnicity is shown in Table 4. There were significant differences among ethnic groups with respect to *cagA* type, pre-EPIYA *cagA* type, *oipA* status, and *jhp0562*/*β*-*(1,3)galT* type (P < 0.001, P = 0.004, P = 0.03, and P = 0.05, respectively). The ABB type was predominant in Papuan strains, and all strains contained the 18-bp deletion. Papuan strains had a low prevalence of the double positive *jhp0562*/*β*-*(1,3)galT* type. Although all Batak’s *H. pylori* were ABD-type *oipA “on”* status, all of the strains contained the 6-bp deletion. Buginese and Dayak ethnicities typically had Western type *cagA* with *oipA “on”* status and no deletion in pre-EPIYA *cagA*. The Batak, Buginese, Chinese, and Floresnese had a higher prevalence of *vacA m2* than the Papuan and Javanese. The Chinese and Floresnese *H. pylori* had a lower prevalence of the *iceA1* genotype than the other ethnicities.

**Table 4:** Association between virulence genes and ethnic groups

Virulence genes	Papuan	Batak	Buginese	Javanese	Chinese	Dayak	Floresnese
Number of strain	9	16	5	2	7	2	3
*cagA* positive	100 %	100 %	100 %	100 %	100 %	100 %	66.7 %
*cagA* type*							
East-Asian-type-*cagA*	11.1 %	100.0 %	40.0 %	0.0 %	71.4 %	0.0 %	100.0 %
Western-type-*cagA*	11.1 %	0.0 %	60.0 %	50.0 %	28.6 %	100.0 %	0.0 %
ABB type	77.8 %	0.0 %	0.0 %	50.0 %	0.0 %	0.0 %	0.0 %
Predominant pre-EPIYA *cagA* type (%)*	18-bp deletion (88.9 %)	6-bp deletion (100.0 %)	No deletion (60.0 %)	No deletion (50.0 %)	39-bp deletion (28.6 %)	No deletion (100.0 %)	18-bp deletion (50.0 %)
*vacA* m2 (%)*	0.0 %	37.5 %	40.0 %	0.0 %	42.9 %	0.0 %	66.7 %
*iceA1* genotype (%)	88.9 %	62.5 %	60.0 %	50.0 %	42.9 %	50.0 %	33.3 %
Double positive *jhp0562*/*β*-*(1,3)galT* (%)*	33.3 %	87.5 %	40.0 %	50.0 %	71.4 %	50.0 %	66.7 %
*oipA* “on” (%)*	77.8 %	100.0 %	100.0 %	100.0 %	100.0 %	50.0 %	66.7 %
*dupA* negative (%)	100.0 %	100.0 %	100.0 %	100.0 %	85.7 %	100.0 %	33.3 %

* P < 0.05

### Nucleotide sequencing

The *cagA* sequencing data for the 38 strains that were *cagA* positive are available under DDBJ accession numbers LC062626 to LC062663. The *oipA* sequencing data of the 39 Indonesian strains are available under DDBJ accession numbers LC062664 to LC062702.

## Discussion

This study complements the results of a previous report [18], in which virulence factors from 5 *H. pylori* strains collected from 1 city in Java island were characterized. Although a small study, this is the first study to characterize the relationship between *H. pylori* virulence factors, ethnicity, and the severity of histological scores. We found that in Indonesian strains, the *cagA* type was predominantly East Asian-type-*cagA*. In comparison to individuals with Western-type-*cagA* strains containing EPIYA-C segments, those infected with East-Asian-type-*cagA* strains containing EPIYA-D segments reported an increased risk of peptic ulcer or gastric cancer [23, 24]. However, surprisingly, we showed that subjects infected with Western-type-*cagA* strains produced more clinical evidence of virulence than those with East-Asian-type-*cagA.* This unusual result could be explained partly by the fact that most East-Asian-type-*cagA* strains contained the pre-EPIYA 6-bp deletion, but not the typical 39-bp deletion. In fact, our data showed that only subjects infected with strains possessing the ABD type-*cagA* containing the 6-bp deletion had significantly lower inflammation and atrophy scores in the corpus than those possessing Western-type-*cagA*. Our previous study showed that pre-EPIYA types appear to be specific for geographic region. No deletion type was predominant in Western countries and the 39-bp deletion type was present in most strains isolated from East Asia. Many Vietnamese strains (75 %) contained the 18-bp deletion, which was rare in other Asian countries [8]. Indonesian strains could not be distinguished from other East Asian strains on the basis of previous genotyping, including the *cagA* repeat region genotypes. Therefore, a unique 6-bp deletion type could be applicable as a new genetic marker for the genomic diversity of *H. pylori* and as a new marker for Indonesian *H. pylori* strains. Indonesia has a lower risk of gastric cancer than Vietnam and East Asian countries such as Japan and South Korea, suggesting that the pre-EPIYA region might have some biological functions that partly contribute to the differences in the incidence of gastric cancer. Further studies will be necessary to investigate the function of the pre-EPIYA region.

There were interesting associations between genotype and ethnicity. The ABB type was predominant in Papuan strains and all strains contained the 18-bp deletion. Papuans are various indigenous peoples of Papua Island and neighboring islands [25]. This ABB type was similar to strain PNGhigh85, which was isolated in Papua (New Guinea) and was classified as hpSahul type by multi-locus sequence typing using seven housekeeping genes [26]. Further studies with a larger sample size are necessary to clarify the association between *H. pylori* genotype and the ethnic groups in Indonesia.

The predominant amino acid sequence of the EPIYA-B motif in the *cagA* genes of Indonesian strains was EPIYT, not EPIYA, for all types of *cagA*. Previous studies reported that EPIYT was the second most common sequence in the EPIYA-B motif of Western-type *cagA*, but was very rare in East-Asian-type-*cagA* [20, 21, 27]. Zhang et al. analyzed 364 Western-type-*cagA* and reported that gastric cancer was associated with the EPIYA sequence in the EPIYA-B motif, whereas the EPIYT sequence was associated with DU [27]. We found that subjects infected with strains with EPIYT sequences had higher activity and inflammation scores in the antrum than those with EPIYA sequences, which is consistent with the association between EPIYT sequences and DU. Antral gastritis induces hyperacidity, which might predispose patients to gastric metaplasia of the duodenal mucosa, which would allow *H. pylori* colonization of the duodenum and further propagate duodenal ulceration [1]. Unfortunately, in the present study we obtained only one patient with DU and could not confirm their results. Contrary to GU, DU has a paradoxical relationship with gastric cancer [28, 29]. Indonesian strains also had a high proportion of the m2 genotype, which was similar to other countries with a low incidence of gastric cancer such as Thailand [30] and Bangladesh [31]. Therefore, the different genotypes of Indonesian *H. pylori* could explain, at least in part, the low incidence gastric cancer in Indonesia. Although gastric carcinogenesis might be influenced by the virulence factors, the host’s genetic and environmental factors also play a role in determining the risk of gastric cancer.

Although the Le antigenic structures were reported to be important for bacterial colonization, adhesion, and evasion of host immune response [32, 33], the role of these in *H. pylori* infection has not been elucidated. Oleastro et al. found the presence of *jhp0562* alone (*jhp0562*-positive/*β*-*(1,3)galT*-negative) was associated with peptic ulcers, rather than with gastritis, and the presence of *β*-*(1,3)galT* alone (*jhp0562*-negative/*β*-*(1,3)galT*-positive) was associated with gastritis, rather than with peptic ulcers [16]. Our previous study revealed that the prevalence of the *jhp0562* and *β*-*(1,3)galT* double positive was significantly higher in strains from the US than in strains from Japan [3]. Moreover, the double positive type was significantly less prevalent in strains from peptic ulcer patients than in those from gastritis patients [3]. The US population has a lower risk of gastric cancer than the Japanese population. Therefore, the predominant double genotypes in Indonesia might be related to the less virulent *H. pylori* strains. Additional in vitro and in vivo studies are necessary to investigate the mechanisms by which these gene products correlate with clinical outcomes. Because these two genes were inversely correlated, the products of the two genes may have the same cell function, thus producing functional redundancy.

Interestingly, only three strains studied were *dupA* positive and there were no intact long-type *dupA*. Schmidt et al. reported there was significant variability in the prevalence of *dupA* among geographical locations, and also among ethnic groups resident in the same country. Indian Malaysia had a low prevalence of *dupA,* which was only 7.1 %, which was lower than in isolates from the Chinese (28.9 %) and the Malay in Malaysia (35.7 %) [34]. It is still unclear why *H. pylori* from some ethnicities lack *dupA*. We previously reported that the intact long-type *dupA* without frameshift mutation, but not the short-type *dupA,* was associated with GU and gastric cancer, but not gastritis, in an Okinawa population in Japan [22]. Therefore, the lack of the *dupA* gene, especially the intact long-type *dupA,* might partly explain the low incidence of gastric cancer in Indonesia.

Our meta-analysis [35] showed that the presence of *iceA1* was associated with peptic ulcer, but not gastric cancer (odds ratio [OR]  =  1.25, 95 % CI  = 1.08–1.44), and that the presence of *iceA2* was inversely associated with peptic ulcer (OR  = 0.76, 95 % CI  =  0.65–0.89). In this study, we also found a significant association between *iceA1* genotypes and peptic ulcer. However, *cagA* [2] and the usage of non-steroidal anti-inflammatory drugs are also important factors for the development of peptic ulcers [36]. To confirm the significance of *iceA*, it is better to perform a multivariate analysis adjusted for the *cagA* status and other risk factors for peptic ulcer. Unfortunately, the number of strains was not sufficient for multivariate analysis. Further studies will be necessary to investigate the association between *H. pylori* virulence factors and peptic ulcers and gastric cancer in Indonesia.

## Conclusions

**Fig. 2:**
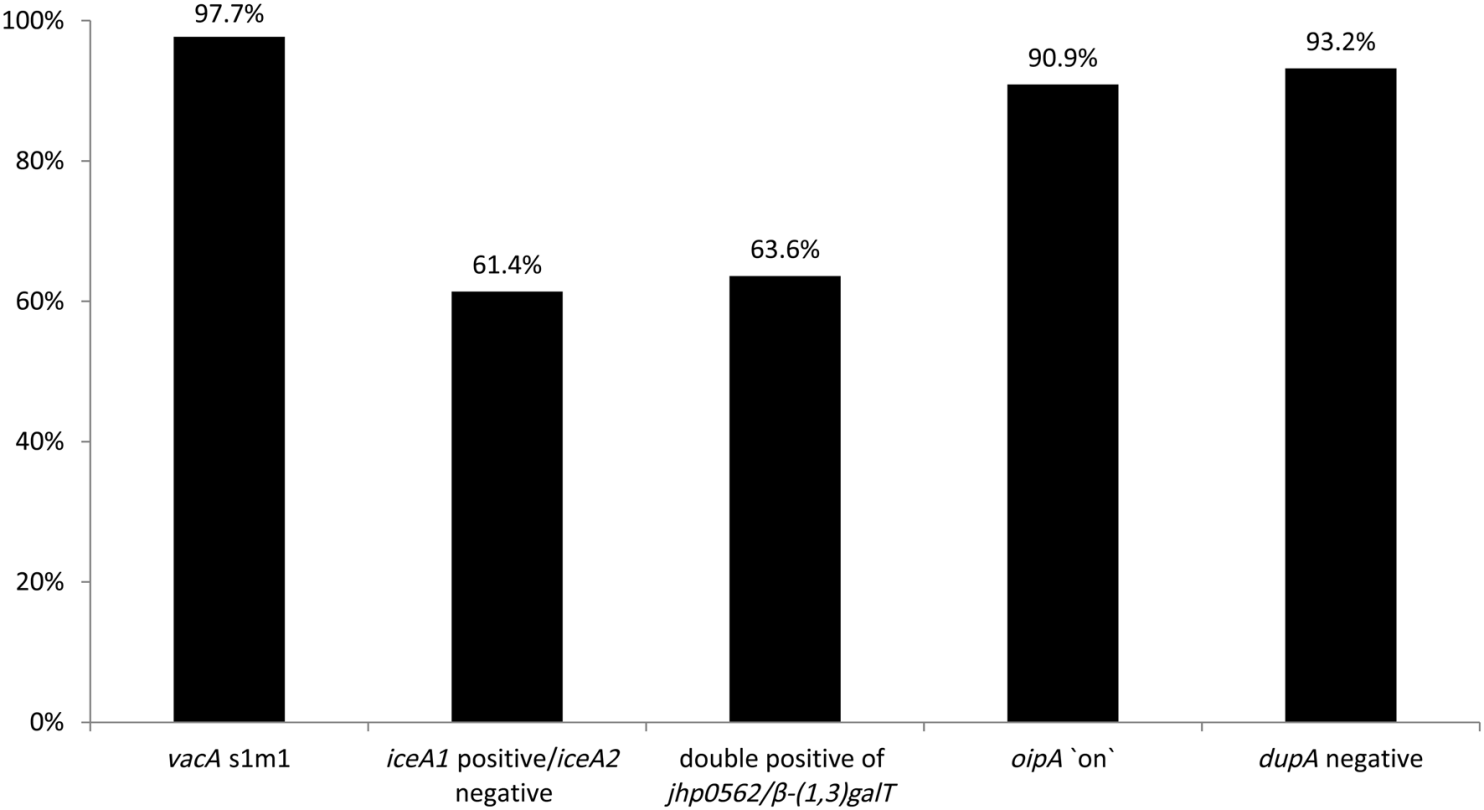
Predominant virulence factors in Indonesian strains. *vacA* s1m1, *iceA1* positive/*iceA2* negative, the double positive for *jhp0562*/*β*-*(1,3)galT*, *oipA* “on”, and *dupA* negative was the predominant genotype in Indonesian strains

Although there are many issues to be confirmed, in addition to low *H. pylori* infection rates, the low incidence of gastric cancer in Indonesia might be attributed to less virulent genotypes of the predominant strains. In general, *cagA* positive (especially East-Asian-type *cagA* containing a 39-bp deletion), *vacA* s1m1, *oipA* “on”, *iceA1* positive, *jhp0562*-positive/*β*-*(1,3)galT*-negative, and intact long-type *dupA* positive are considered to be virulent genotypes [37]. In contrast, we found that the predominant genotypes in Indonesian strains included East-Asian-type-*cagA* containing a 6-bp deletion, m2, *dupA* negative/short type *dupA*, and the *jhp0562*/*β*-*(1,3)galT* double positive genotypes (Fig. 2).

## Methods

Gastric biopsy specimens were taken from the antrum (pyloric gland area) and the corpus (fundic gland area). Biopsy specimens for culture were immediately placed at −20 °C and stored at −80 °C within a day of collection until they were used for culture testing. Two antral specimens were used for *H. pylori* culture and histological examination. One corporal specimen was used for histological examination. Patients were considered to be negative for *H. pylori* infection when culture and histology results were negative, whereas patients with at least one positive test result were considered positive for *H. pylori* infection. Written informed consent was obtained from all participants, and the study protocol was approved by the Ethics Committee of Dr. Cipto Mangunkusumo Teaching Hospital (Jakarta, Indonesia), Dr. Soetomo Teaching Hospital (Surabaya, Indonesia), Dr. Wahidin Sudirohusodo Teaching Hospital (Makassar, Indonesia), and Oita University Faculty of Medicine (Yufu, Japan).

### Determination of gastritis stage

All biopsy materials for histological testing were fixed in 10 % buffered formalin and embedded in paraffin. Serial sections were stained with hematoxylin and eosin as well as May–Giemsa stain. The degree of inflammation, neutrophil activity, atrophy, intestinal metaplasia, and bacterial density were classified into 4 grades according to the updated Sydney system: 0, ‘normal’; 1, ‘mild’; 2, ‘moderate’; and 3, ‘marked’ [38]. Samples with grade 1 or more atrophy were considered atrophy-positive [39]. Immunohistochemistry was performed as previously described [40]. Briefly, after antigen retrieval and inactivation of endogenous peroxidase activity, tissue sections were incubated with α-*H. pylori* antibody (DAKO, Denmark) overnight at 4 °C. After washing, the sections were incubated with biotinylated goat antirabbit IgG (Nichirei Co., Japan), followed by incubation with an avidin-conjugated horseradish peroxidase solution (Vectastain Elite ABC kit; Vector Laboratories Inc., Burlingame, CA, USA). Peroxidase activity was detected using an H_2_O_2_/diaminobenzidine substrate solution.

### *H. pylori* isolation and genotyping

*H. pylori* colonies were cultured from antral biopsy specimens using standard methods [9]. For *H. pylori* culture, 1 antral biopsy specimen was homogenized in saline and inoculated onto Skirrow’s medium and incubated for up to 10 days at 37 °C under microaerophilic conditions (10 % O_2_, 5 % CO_2_, and 85 % N_2_). *H. pylori* were identified on the basis of colony morphology, Gram staining results, and positive reactions for oxidase, catalase, and urease. Isolated strains were stored at −80 °C in Brucella Broth (Difco, NJ, USA) containing 10 % dimethyl sulfoxide and 10 % horse serum. *H. pylori* DNA was extracted from these colonies for *H. pylori* genotyping using the QIAamp DNA Mini Kit (QIAGEN, Valencia, CA, USA) according to the manufacturer’s directions. The list of primers used for the detection of virulence factors of *H. pylori* is shown in Table 5. The *cagA* status was determined by polymerase chain reaction (PCR) amplification and direct sequencing of the EPIYA repeat region and the pre-EPIYA region. The *oipA* status was determined by PCR-based sequencing of the signal region, as described previously [8, 24, 41]. The presence of the *vacA* genotype (s1 or s2, and m1 or m2), *iceA* (*iceA1* or *iceA2*), *dupA, jhp0562*, and *β*-*(1,3)galT* were determined based on PCR product size, as described previously [22, 42–45]. The amplified fragment was detected by 1.5 % agarose gel electrophoresis. DNA sequencing was performed using a Big Dye Terminator v3.1 Cycle Sequencing Kit and an AB 3130 Genetic Analyzer (Applied Biosystems, Foster City, CA, USA), according to the manufacturer’s instructions. Multiple sequence alignments of the *cagA* pre-EPIYA and *cagA* were generated using MAFFT version 7 (available at http://mafft.cbrc.jp/alignment/server/) and confirmed by visual inspection.

**Table 5:** The primers used for detecting virulence factors of *H. pylori*

Genes	Primer sequences (5′ → 3′)	PCR product (bp)	PCR conditions
*cagA*	ACC CTA GTC GGT AAT GGG	521	94 °C, 1 min; 52 °C, 1 min; 72 °C, 1 min (35 cycles)
	GCT TTA GCT TCT GAY ACY GC^a^		
*vacA*			94 °C, 1 min; 52 °C, 1 min; 72 °C, 1 min (35 cycles)
s1/s2	ATG GAA ATA CAA CAA ACA CAC	259/268	
	CTG CTT GAA TGC GCC AAA C		
m1/m2	CAA TCT GTC CAA TCA AGC GAG	567/642	
	GCG TCA AAA TAA TTC CAA GG		
*oipA*	CCA TGA AAA AAG CTC TCT TAC T	430	94 °C, 30 s; 50 °C, 30 s; 72 °C, 30 s (25 cycles)
	GCC CTT TTA CCC TTC GTT CAA C		
*iceA*			94 °C, 1 min; 56 °C, 1 min; 72 °C, 1 min (30 cycles)
*iceA1*	GTG TTT TTA ACC AAA GTA TC	247	
	CTA TAG CCA STY TCT TTG CA		
*iceA2*	GTT GGG TAT ATC ACA ATT TAT	229	
	TTT CCC TAT TTT CTA GTA GGT		
*dupA* F2R2	ATG TTT CTT GGT TTA GAG GG	2499	95 °C, 30 s; 56 °C, 30 s; 72 °C, 30 s (35 cycles)
	TTA TAC ATA TTG AAT ATT CTC GC		
*dupA* F5R3	GGT TTC TAC TGA CAG AGC GC	468	
	CGT ATT TAG TCA GTA AGT TGG CG		
*jhp0562*/*β*-*(1,3)galT*	TGA AAA GCC CTT TTG ATT TTG	301/602	95 °C, 30 s; 56 °C, 30 s; 72 °C, 30 s (35 cycles)
	GCT GTA GTG GCC ACA TAC ACG		

^a^Y = C + T

### Statistical analysis

Data were analyzed using SPSS, version 19 (SPSS Inc., Chicago, IL, USA). Discrete variables were tested using the Chi square test; continuous variables were tested using Mann–Whitney *U* and *t*-tests. A two-tailed *P* value ≤0.05 was considered statistically significant.

## Abbreviations

PCR: polymerase chain reaction
SHP2: Src homology-2 domain-containing phosphatase 2
IL: interleukin
DU: duodenal ulcer
LPS: lipopolysaccharide
Le: lewis
GU: gastric ulcer
